# Does mandatory reporting legislation increase contact with child protection? – a legal doctrinal review and an analytical examination

**DOI:** 10.1186/s12889-018-5864-0

**Published:** 2018-08-16

**Authors:** Lil Tonmyr, Ben Mathews, Margot E. Shields, Wendy E. Hovdestad, Tracie O. Afifi

**Affiliations:** 1 Public Health Agency of Canada, 785 Carling Ave, AL 6807B, Ottawa, ON K1A 0K9 Canada; 2 Australian Centre for Health Law Research, School of Law, Faculty of Law, Queensland University of Technology, 2 George St, Brisbane, QLD 4000 Australia; 3 Department of Community Health Sciences, Department of Psychiatry, University of Manitoba, Winnipeg, MB R3T 2N2 Canada

**Keywords:** Child maltreatment, Exposure to intimate partner violence, Sexual abuse, Physical abuse, Mandatory reporting, Child welfare, Doctrinal legal review

## Abstract

**Background:**

Within Canadian provinces over the past half-century, legislation has been enacted to increase child protection organization (CPO) involvement in situations of child maltreatment (CM). This study had two objectives: 1) to document enactment dates of legislation for mandatory reporting of CM; 2) to examine reported CPO involvement among people reporting a CM history in relation to the timing of these legislative changes.

**Methods:**

The history of mandatory reporting of CM was compiled using secondary sources and doctrinal legal review of provincial legislation. The 2012 Canadian Community Health Survey - Mental Health (CCHS-MH) with *n* = 18,561 was analyzed using birth cohorts to assess associations between the timing of legislation enactment and contact with CPO.

**Results:**

All Canadian provinces currently have mandatory reporting of physical and sexual abuse; 8 out of 10 provinces have mandatory reporting for children’s exposure to intimate partner violence. Increases in reporting CM to CPOs paralleled these laws’ enactment, particularly for severe and frequent CM.

**Conclusions:**

These findings show that mandatory reporting laws increase reporting contact with CPO, particularly for severe and frequent CM. Whether they have had the intended effect of improving children’s lives remains an important, unanswered question.

## Background

In Canada today, child maltreatment (CM) occurs in every stratum of society. The negative impacts pose serious immediate and long-term risks to the health and development of its victims [1]. CM impedes healthy development and negatively impacts mental and physical health [1]. It also increases the likelihood of unhealthy behaviors, such as smoking and substance use [2–4]. CM and associated problems are threats to public health.

CM is by no means a rare occurrence. Results from the Canadian Community Health Survey-Mental Health Survey (CCHS-MH) conducted in 2012 indicate that at least 32% of the adult Canadian population experienced one or more types of childhood maltreatment [5]. Childhood physical abuse (CPA) was the most common form of CM—reported by 26% of CCHS-MH respondents, followed by sexual abuse (CSA), reported by 10%, and exposure to intimate partner violence (CEIPV), by 8% [5]. Neglect and emotional maltreatment were not captured in the 2012 CCHS-MH.

Within a few decades of Canada’s confederation, child protection organizations (CPOs) were introduced. The inception and development of CPOs occurred at different times across the provinces. The first general frameworks for child welfare were established in 1893 in Ontario, in 1902 in Manitoba, and 1906 in Nova Scotia [6]. While the initial focus was primarily on preventing neglected children from having to subsist in the streets, advances in the 1960s and 1970s broadened systemic welfare strategies [7]. One such development was the implementation of laws requiring reporting of CM, similar to those introduced in the 1960s in the United States. These laws were enacted in the provinces at different times and varied in content [7]. Mandatory legislation has been successfully implemented as a public health strategy in other domains such as the requirement for immunizations for children attending school [8].

Despite mandatory reporting legislation, evidence suggests that only a small share of CM is brought to the attention of CPOs. Based on a study of the population aged 15 years or older conducted in the province of Ontario in 1990/1991 [9], reports of contact with a CPO were 5.1% among those with a history of CPA, and 8.7% among those with a history of CSA. A similar study using data collected in the 10 Canadian provinces from the 2012 CCHS-MH [10] indicated that the likelihood of CPO contact was slightly higher, 8.0% among those with a history of CPA, 10.4% among those with a history of CSA and 16.6% among those with a history of CEIPV (CEIPV was not captured in the earlier study conducted in Ontario). Among those with a history of all three types of CM (CPA, CSA and CEIPV), contact was reported by 24.5%.

Although little is known about the effectiveness of CPO interventions in deterring CM, and improving health and social outcomes [1], it is important to understand how legislative changes may influence reporting practices. The objectives of this study are: 1) to examine the history of legislative changes and the key elements of CM mandatory reporting laws in Canada; and 2) to examine the likelihood of reporting contact with a CPO among people reporting a history of CM in relation to the date of enactment of mandatory reporting legislation. The higher reporting rates observed in the study based on the 2012 CCHS-MH [10] compared with the earlier Ontario study from 1990 [9] suggests that the likelihood of CM being reported to CPOs has increased over time. To explore this hypothesis, we examine reporting rates by age cohorts among CCHS-MH respondents.

## Methods

### Doctrinal legal review

Doctrinal legal research can involve a literature review but also requires a trained expert in legal doctrine to read and analyse the primary sources of law [11]. Our analysis of the law was necessarily focused on legislation as a primary source, since child protection is regulated by legislation rather than case law. Any judicial decisions about the legislation would have added little useful information. An initial scan of the legislative history and framework in each province was undertaken by identifying secondary source coverage of child protection legislation in legal and social science databases [7, 12–14]. Then, the actual legislation for each province was reviewed, using electronic legislation databases and orthodox legal analysis using principles of statutory interpretation. Because electronic databases do not contain records of legislation from the 1960s, a hand search was carried out in August 2016 in a specialized law library for hard copies of legislation for each province. This strategy enabled identification of the relevant legislation.

Analysis of multiple provisions in each piece of legislation then enabled identification of the mandatory reporting duty and its wording and scope, the definition of key concepts such as “abuse”, “neglect” and “child in need of protection,” and the commencement date of the legislation. That is, multiple relevant parts of each statute were analyzed to identify the discrete provisions in each that detail the reporting duty, and associated provisions which define relevant terms which further establish and elucidate the nature and scope of the duty. In a number of cases, it was not possible to definitively identify subsequent amending legislation and its precise commencement date, although this generally applied to jurisdictions with small populations (i.e., Newfoundland and Labrador, Prince Edward Island). Where this occurred, triangulation of the analysis with conclusions drawn from secondary sources was used to confirm the interpretation.

It is noteworthy that legal provisions are often ambiguous, and while reporting laws were generally unequivocal, questions arose for some jurisdictions about whether the reporting duty applied to CSA. In such instances, conventional technical principles of statutory interpretation were applied to draw conclusions, based on the legislation’s text, context, and purpose.^1^

### CCHS data set and analysis

#### CCHS data

The 2012  CCHS-MH [15] was used to address the question of whether the enactment of laws requiring mandatory reporting of CM was associated with changes in the likelihood of CPO involvement in cases of CM. The CCHS-MH was developed by Statistics Canada in collaboration with Health Canada, the Public Health Agency of Canada, provincial health ministries, an expert advisory group, and academic experts.

The target population for the 2012 CCHS-MH comprised household residents aged 15 or older living in the 10 Canadian provinces. Excluded from the survey’s coverage were persons living on reserves and other Aboriginal settlements, full-time members of the Canadian Forces and the institutionalized population. Due to the nature of the questions asked in the CCHS-MH, proxy responses were not permitted making it necessary to exclude the institutionalized population. There are complexities regarding obtaining permission to conduct in-person interviews on military sites and reserves and therefore people living in these jurisdictions were excluded. Another reason to exclude the Canadian Forces is that they have a distinct health system and a separate health survey. Altogether, these exclusions represent about 3% of the target population. The response rate was 68.9%, yielding a sample of 25,113 individuals aged 15 or older [15]. This analysis is based on the “share” file (*n* = 23,709; 94%), a subset of the sample consisting of the records of respondents who agreed that their information could be shared with Statistics Canada’s partners. The majority of interviews (87%) were conducted in person using computer assisted interviewing.

##### Inclusion and exclusion criteria

The questions on CM were asked of respondents aged 18 or older (*n* = 22,486). Immigrants to Canada were excluded from the analysis (since it was not determined if CM occurred before or after immigration to Canada), reducing the sample size for this study to 18,561. Non-response to the individual questions on CM ranged from 0.9 to 1.2%; non-response to the item on contact with a CPO was 0.3%.

##### Informed consent

Respondents were informed about privacy, confidentiality and voluntary participation for the survey and provided informed consent prior to their participation [16].

#### Measures

##### CM variables

**Fig. 1:**
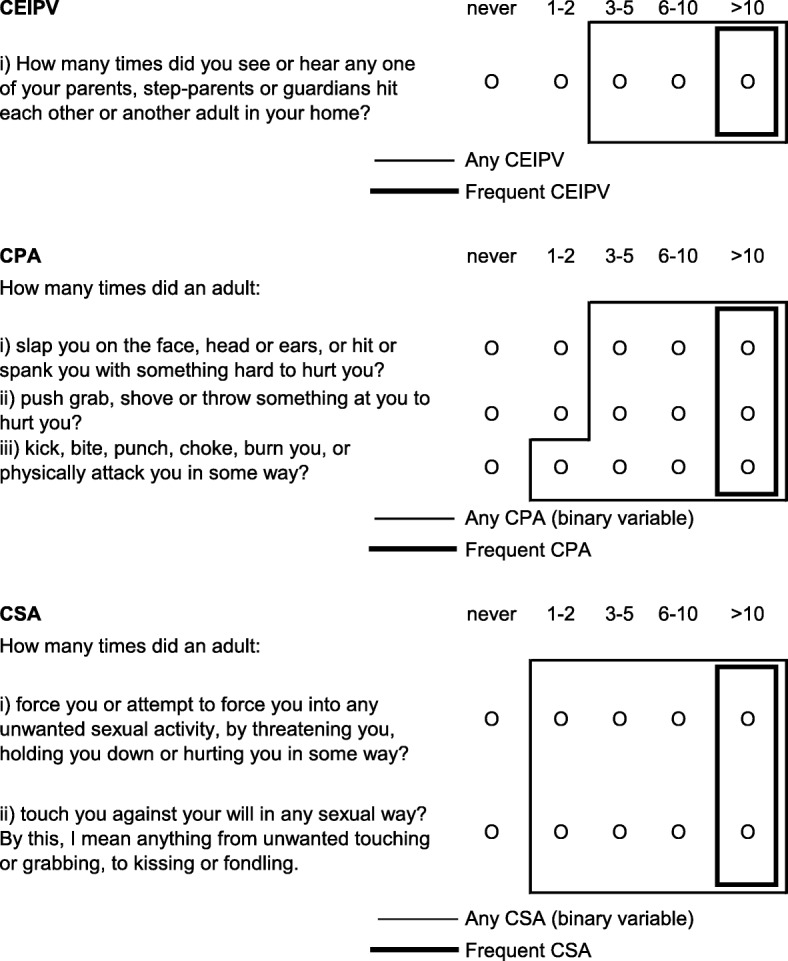
Child maltreatment items and definitions CPA = Childhood physical abuse CSA = Childhood sexual abuse CEIPV=Childhood exposure to intimate partner violence

The occurrence of CPA, CSA and CEIPV was assessed by asking respondents about specific experiences (“*things that may have happened to you before you were 16 in your school, in your neighbourhood, or in your family*”) (Fig. 1). The source of the items for CPA and CEIPV is the Childhood Experiences of Violence Questionnaire (CEVQ) [17]. The CSA items are very similar to those used in the 2009 General Social Survey [18]. For each type of abuse, binary variables (yes/no) were created following CEVQ guidelines [17]. *Contact with a child protection organization (CPO)* was determined with the question, “Before age 16, did you ever see or talk to anyone from a child protection organization about difficulties at home?”

##### Socio-demographic variables

The socio-demographic characteristics used as controls in logistic regression models included, *sex, respondent’s highest level of education* (less than secondary graduation, secondary graduation, some postsecondary, postsecondary graduation), *household income* (quintiles based on household income adjusted by Statistics Canada’s low income cutoffs (LICO) specific to the number of individuals in the household, the size of the community, and the survey year), *ethnicity* (White, non-White) and *province of residence* at the time of the survey.

#### Analysis

Among individuals reporting CM, cross-tabulations were used to examine associations between year of birth and reporting contact with a CPO. Cohorts based on year of birth were established to examine any difference in the percentage of people reporting contact with a CPO before and after 1965—the year mandatory reporting was first implemented in Canada (in the province of Ontario). The following *detailed* birth cohorts were defined by year of birth:

- 1939 or earlier (age > = 26 years in 1965)
- 1940–1949 (age 16–25 years in 1965)
- 1950–1957 (age 8–15 years in 1965)
- 1958–1965 (age 0–7 years in 1965)
- 1966–1974 (born 1–9 years after 1965 legislation)
- 1975–1984 (born 10–19 years after 1965 legislation)
- 1985–1994 (born 20–29 years after 1965 legislation)

In people reporting any type of CM within each of these *detailed* birth cohorts, the percentage reporting contact with a CPO was estimated. We also examined percentages reporting CPO contact for specific types of CM but sample sizes were too small and therefore, to enlarge the cell sizes and thereby increase the stability of CM-specific estimates, the birth cohorts were more *broadly* defined. These analyses focused on CPO contact rates among respondents born after 1965 (when mandatory reporting was introduced in Canada), but before or during the year mandatory reporting was implemented in the respondent’s province of residence at the time of the survey. The *broad* birth cohorts derived for estimates pertaining to specific types of CM were:

- born before or during 1965
- born after 1965, but before or during the year mandatory reporting was implemented in current province of residence
- born after the year mandatory reporting was implemented in current province of residence.

For this broader categorization, cross-tabulations and logistic regression (controlling for selected socio-demographic characteristics) were used to examine associations between year of birth and contact with a CPO among people reporting specific types of CM, as well as frequency and severity of CM.

Analyses were conducted using SAS Enterprise Guide 5.1. All estimates were based on weighted data. Weights were created at Statistics Canada so that the data would be representative of the Canadian population living in the ten provinces in 2012 and were adjusted to compensate for non-response. Variance estimates and 95% confidence intervals (CIs) were calculated using the bootstrap technique (with the SAS “proc survey” procedures) to account for the complex survey design of the CCHS-MH [15].

## Results

### Doctrinal legal review

Doctrinal and historical analysis of provincial legislation indicated that duties to report CPA and CSA of children have been enacted in each province, but at different times. The first mandatory reporting laws appeared in Ontario in 1965, followed by Alberta in 1966, and other jurisdictions soon thereafter. The initial laws focused predominantly on child neglect, CPA or ill-treatment, and other indications of being “in need of protection,” which itself was variously defined. Duties to report CSA have also been enacted, sometimes explicitly, or else by implication. At this early stage, no jurisdiction expressly acknowledged CEIPV, nor would it have been incorporated by implication.

The area of greatest variation pertained to the duty to report CEIPV. At the time of writing, eight of the ten provinces expressly required reports of exposure of a child to domestic violence or intimate partner violence. The timing of the introduction of this duty varied substantially, from 1973 in Saskatchewan until most recently in June 2014. Three jurisdictions (i.e., New Brunswick and Ontario) still do not explicitly require reports of CEIPV.

The four most populous Canadian jurisdictions, Alberta, British Columbia, Ontario, and Quebec, account for over 85% of the national population [19]. Three of these jurisdictions - Alberta, British Columbia, and Ontario - enacted mandatory reporting duties at around the same time in 1965 to 1967, with Quebec following almost a decade later. In these jurisdictions, the legislation pertaining to CPA and CSA is similar, although not for CEIPV. The scope of the legislation and commencement dates of the mandatory reporting duties for each type of maltreatment in the ten provinces is summarized in Table 1, below.

**Table 1:** Scope and commencement dates of legislative mandatory reporting duties for physical abuse, sexual abuse, and exposure to intimate partner violence: Canadian provinces

Jurisdiction	Physical abuse	Sexual abuse	Exposure to domestic violence (or family violence, or intimate partner violence)
Alberta	7 April 1966	7 April 1966	31 May 1984
British Columbia	23 March 1967	23 March 1967	1 June 2014
Manitoba	10 June 1974	10 June 1974	At latest by 1 January 2003^a,b^
New Brunswick	16 July 1980	16 July 1980	Not mandated
Newfoundland & Labrador	5 May 1972	5 May 1972	1981^a^
Nova Scotia	20 May 1976	20 May 1976	19 June 1990
Ontario	22 June 1965	22 June 1965	Not mandated
Prince Edward Island	24 April 1981	24 April 1981	At latest by 1 November 2003^c^
Quebec	28 December 1974	28 December 1974	9 July 2007
Saskatchewan	27 April 1973	27 April 1973	27 April 1973

^a^Unable to pinpoint commencement date: year/time indicated is based on secondary sources [42] and analysis of available legislative materials

^b^While the statute did not expressly apply to exposure to domestic violence, its broad provisions read together produced a legitimate interpretation that the duty applied to this exposure, and that this accorded with the understanding of stakeholders

^c^s 3(f) of the definition of “child in need of protection” in the Child Protection Act version current to 1 November 2003, and s 22 mandatory reporting duty applying to a “child in need of protection” (electronic version of Act on CanLII current to this date). A search of PEI Hansard indicates no identifiable discussion of legislative developments prior to this date

### Statistical analysis of CCHS-MH data

#### CPO contact by detailed birth cohorts

**Fig. 2:**
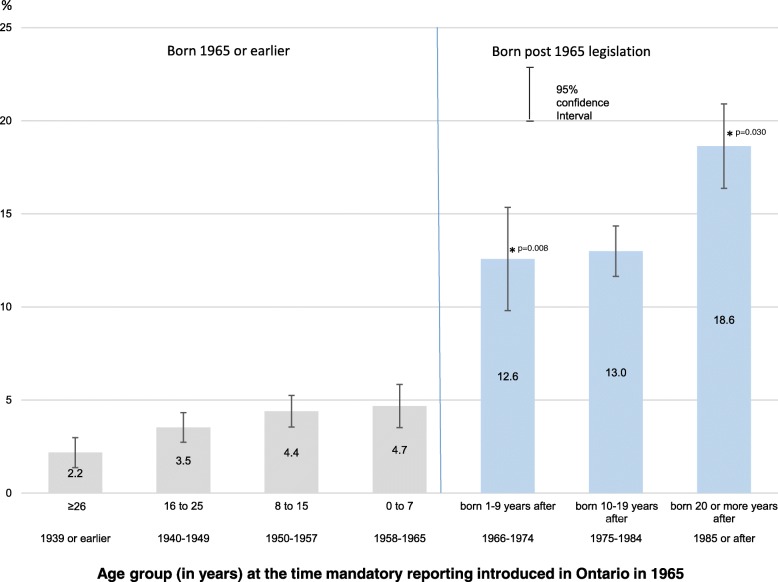
Percent reporting seeing/talking to someone from a child protection organization about difficulties at home by age group (birth cohort) at the time mandatory reporting introduced in Ontario in 1965, household population aged 18 or older, excluding immigrants to Canada in 2012 * Significantly higher than previous age group

Among non-immigrants who reported CM, 8.6% reported having had contact with a CPO. In our analysis of detailed birth cohorts, percentages reporting contact with CPO (Fig. 2) were very low (< 5%) for cohorts born before/during 1965. For those born 1 to 9 years after the 1965 legislation, CPO contact increased sharply, with 12.6% reporting CPO involvement. Further increases were observed for younger cohorts, with 18.6% reporting CPO involvement among those born 20 or more years after the 1965 legislation.

#### CPO contact by broad birth cohorts

Table 2 presents the results when CPO involvement was examined using the more broadly defined cohorts in relation to age when mandatory reporting was first introduced in Canada as well as the respondent’s current province of residence. Among people born before or in 1965 who reported a history of CM, only 4.1% reported CPO involvement. This percentage increased to 8.6% among those born after 1965, but before or during the year mandatory reporting was first introduced in the current providence of residence, and to 15.3% among those born after mandatory reporting was introduced in the current province of residence.

**Table 2:** Percent reporting seeing/talking to someone from a child protection organization about difficulties at home by year of birth (birth cohort) when mandatory reporting introduced in Canada or Current Province of Residence, Household Population^c^ Aged 18 or Older, 2012

% among those reporting:	Total		Year of birth								
			Estimate 1		Estimate 2			Estimate 3			
			Born before/during the year mandatory reporting first introduced in Canada (i.e., 1965 or earlier)		Born before/during the year mandatory reporting first introduced in current province of residence but after 1965			Born after the year mandatory reporting first introduced in current province of residence			
	%	95% CI	%	95% CI	%	95% CI	Difference between Estimate 2 and Estimate 1	%	95% CI	Difference between Estimate 3 and Estimate 2	Difference between Estimate 3 and Estimate 1
Any childhood maltreatment	8.6	(7.3, 9.9)	4.1	(3.0, 5.1)	8.6	(4.0, 13.1)^b^	4.5	15.3	(12.3, 18.3)^a^	6.7	11.2
Childhood physical abuse (CPA)	9.1	(7.5, 10.8)	4.3	(3.0, 5.6)	11.0	(4.8, 17.1)^b^	6.7	15.4	(12.0, 18.8)^b^	4.4	11.1
Slapped on face head or ears, hit or spanked with something hard	9.7	(7.9, 11.6)	4.6	(3.1, 6.1)	13.0	(5.7, 20.3)^b^	8.4	16.0	(12.3, 19.8)^b^	3.0	11.4
Pushed, grabbed, shoved, something thrown at	16.5	(13.0, 20.0)	7.6	(4.8, 10.4)	19.3	(8.2, 30.4)^b^	11.7	27.8	(20.7, 34.9)^b^	8.5	20.2
Kicked, bit, punched, choked, burned, attacked	17.0	(13.3, 20.8)	8.4	(5.4, 11.4)	19.4	(7.1, 31.6)	10.9	28.2	(20.6, 35.8)^b^	8.9	19.8
Childhood sexual abuse (CSA)	11.3	(9.2, 13.4)	6.2	(4.1, 8.3)	8.2	(3.0, 13.4)	2.0	24.1	(18.9, 29.3)^a^	15.9	17.9
forced (attempted forced) unwanted sexual activity	14.3	(11.3, 17.3)	9.2	(5.7, 12.7)	15.9	(5.7, 26.2)	6.7	24.6	(17.8, 31.3)^b^	8.7	15.4
unwanted sexual touching	11.8	(9.6, 14.0)	6.5	(4.2, 8.8)	8.4	(3.1, 13.8)	1.9	24.5	(19.2, 29.9)^a^	16.1	18.0
Childhood exposure to intimate partner violence (CEIPV)	19.8	(15.4, 24.1)	9.2	(5.6, 12.7)	14.2	(5.9, 22.6)	5.1	34.1	(25.0, 43.1)^a^	19.8	24.9
Number of childhood maltreatment types											
1 type	4.7	(3.7, 5.6)	2.3	(1.4, 3.2)	6.4	(0.8, 12.0)	4.1	7.9	(5.9, 9.8)^b^	1.5	5.6
2 types	14.2	(10.2, 18.3)	5.1	(3.2, 7.1)	11.8	(5.6, 18.1)	6.7	28.0	(18.4, 37.5)^a^	16.1	22.8
3 types	27.5	(20.3, 34.6)	16.6	(7.8, 25.3)	22.6	(3.7, 41.6)	6.1	47.8	(35.0, 60.7)^a^	25.2	31.3
More than ten times											
For any childhood abuse question	13.5	(10.8, 16.3)	6.8	(4.5, 9.2)	19.3	(8.8, 29.8)	12.4	21.6	(15.8, 27.4)^b^	2.3	14.7
Slapped on face head or ears, hit or spanked with something hard	13.6	(10.4, 16.8)	5.5	(3.6, 7.5)	21.2	(8.4, 33.9)^b^	15.6	23.0	(16.1, 29.9)^b^	1.9	17.5
Pushed, grabbed, shoved, something thrown at	20.9	(15.0, 26.8)	8.0	(5.0, 10.9)	29.5	(10.7, 48.3)	21.5	36.4	(23.8, 48.9)^b^	6.9	28.4
Kicked, bit, punched, choked, burned, attacked	27.7	(18.0, 37.4)	11.6	(7.0, 16.3)	38.1	(11.4, 64.8)	26.5	43.9	(25.2, 62.6)^b^	5.8	32.3
Forced/attempted forced unwanted sexual activity	26.2	(17.3, 35.1)	18.1	(10.7, 25.5)	31.1	(7.9, 54.3)	13.0	40.9	(17.8, 63.9)	9.7	22.8
Unwanted sexual touching	21.6	(14.6, 28.7)	15.9	(9.8, 21.9)	26.9	(9.9, 44.0)	11.1	30.8	(13.3, 48.3)	3.9	14.9
Exposure to intimate partner violence	22.9	(15.8, 29.9)	11.0	(5.6, 16.5)	22.0	(8.0, 35.9)	10.9	38.4	(23.6, 53.3)^b^	16.5	27.4
No childhood maltreatment reported	1.9	(1.4, 2.4)	1.1	(0.7, 1.5)	0.7	(0.2, 1.3)	−0.4	2.9	(1.9, 3.9)^a^	2.2	1.8

Source: Statistics Canada, Canadian Community Health Survey -- Mental Health, 2012 (share file)

*CI* Confidence interval

^a^Significantly higher than Estimate 2

^b^Significantly higher than Estimate 1

^c^Excluding immigrants to Canada

#### CPO contact by broad birth cohorts for specific types of CM

A similar pattern emerged between birth year and CPO contact for the specific types of maltreatment. The sharpest increases in rates of CPO contact were observed in those reporting more than one type of CM and for more severe forms of CM. For example, among respondents who reported all three types of CM (CPA, CSA and CEIPV), 16.6% of those born before or during 1965 reported CPO involvement. Among those born after the year mandatory reporting legislation was introduced in their current province of residence, close to half (47.8%) reported CPO involvement. Among respondents who reported being kicked, bitten, punched, choked, burned or attacked more than 10 times, CPO involvement rates increased from 11.6% for those who were born before or during 1965 to 43.9% for those born after the year mandatory reporting legislation was introduced in their current province of residence. Although sample counts were low for those reporting frequent abuse (as indicated by the wide confidence intervals), the sharp increases in rates observed for all CM types provides compelling evidence that the legislation had an impact.

#### CPO contact by those reporting no CM

As expected, reporting contact with a CPO was rare (1.9%) among those with no history of the three measured types of CM. However, the rate was slightly higher (2.9%) for those born after mandatory reporting was introduced in the current province of residence compared with those born before/during 1965 (1.1%).

#### Results of logistic regressions

Among those reporting CM, associations between the broadly defined birth cohorts and reporting contact with a CPO were examined in logistic regression models. To examine the possible impact of socio-demographic factors on the magnitude of associations, unadjusted odds were calculated first, and then the control variables were included in the models. The odds of reporting CPO involvement were consistently higher among those born after the year mandatory reporting legislation was introduced in their current province of residence. For example, among respondents reporting any CM, in the fully adjusted model, those who were born after the year mandatory reporting legislation was introduced in their current province of residence had 4.2 times the odds of reporting CPO involvement compared with those born before/during the year mandatory reporting was first introduced in Canada; among those reporting all three types of CM the corresponding odds were 5.4 times higher (Table 3).

**Table 3:** Unadjusted and adjusted^a^ odds ratios for reporting seeing/talking to someone from a child protection organization about difficulties at home by age group at the time mandatory reporting introduced in Ontario in 1965/first introduced in current province of residence, household population^b^ aged 18 or older, 2012

	Year of birth								
	Born before/ during the year mandatory reporting first introduced in Canada (i.e., 1965 or earlier)	Born before/during the year mandatory reporting first introduced in current province of residence but after 1965				Born after the year mandatory reporting first introduced in current province of residence			
		Unadjusted Odds		Adjusted Odds		Unadjusted Odds		Adjusted Odds	
Odds among those reporting:	(reference)	Odds	95% CI	Odds	95% CI	Odds	95% CI	Odds	95% CI
Any childhood maltreatment		2.2*	(1.2, 4.2)	2.3*	(1.2, 4.4)	4.3**	(3.0, 6.1)	4.2**	(2.9, 6.1)
Childhood physical abuse (CPA)		2.7**	(1.4, 5.5)	3.2**	(1.5, 6.7)	4.0**	(2.7, 6.1)	4.0**	(2.6, 6.2)
Slapped on face head or ears, hit or spanked with something hard		3.1**	(1.5, 6.3)	3.8**	(1.8, 8.2)	3.9**	(2.5, 6.1)	4.0**	(2.5, 6.3)
Pushed, grabbed, shoved, something thrown at		2.9*	(1.3, 6.6)	3.7**	(1.5, 9.0)	4.7**	(2.8, 7.8)	4.2**	(2.6, 7.0)
Kicked, bit, punched, choked, burned, attacked		2.6*	(1.0, 6.5)	3.4*	(1.3, 9.2)	4.3**	(2.5, 7.2)	3.9**	(2.4, 6.4)
Childhood sexual abuse (CSA)		1.3	(0.6, 2.9)	1.3	(0.6, 2.8)	4.8**	(3.0, 7.6)	4.1**	(2.5, 6.7)
forced (attempted forced) unwanted sexual activity		1.9	(0.8, 4.5)	1.7	(0.7, 4.4)	3.2**	(1.8, 5.7)	2.6**	(1.5, 4.8)
unwanted sexual touching		1.3	(0.6, 2.9)	1.3	(0.6, 2.8)	4.7**	(2.9, 7.5)	4.0**	(2.4, 6.7)
Childhood exposure to intimate partner violence (CEIPV)		1.6	(0.7, 3.8)	1.9	(0.7, 4.9)	5.1**	(2.8, 9.3)	5.3**	(3.0, 9.5)
Number of childhood maltreatment types									
1 type		2.9	(1.0, 8.6)	2.6	(0.9, 7.9)	3.6**	(2.2, 5.9)	3.7**	(2.2, 6.2)
2 types		2.5*	(1.2, 5.0)	2.5*	(1.1, 5.4)	7.2**	(3.9, 13.3)	6.4**	(3.6, 11.4)
3 types		1.5	(0.3, 6.3)	1.8	(0.4, 7.3)	4.6**	(2.0, 10.6)	5.4**	(2.1, 13.6)
More than ten times									
For any childhood abuse question		3.2**	(1.5, 7.0)	4.0**	(1.7, 9.4)	3.7**	(2.3, 6.2)	4.0**	(2.4, 6.8)
Slapped on face head or ears, hit or spanked with something hard		4.6**	(1.9, 11.0)	5.7**	(2.0, 16.1)	5.1**	(3.0, 8.9)	5.9**	(3.2, 10.8)
Pushed, grabbed, shoved, something thrown at		4.8**	(1.7, 13.6)	6.0**	(1.8, 20.6)	6.6**	(3.4, 12.9)	6.2**	(3.2, 12.1)
Kicked, bit, punched, choked, burned, attacked		4.7*	(1.2, 18.1)	10.1**	(1.9, 52.8)	5.9**	(2.4, 14.4)	6.1**	(2.3, 16.3)
Forced/attempted forced unwanted sexual activity		2.0	(0.6, 7.6)	2.5	(0.5, 14.1)	3.1*	(1.0, 9.5)	2.4	(0.7, 8.1)
Unwanted sexual touching		2.0	(0.7, 5.3)	1.8	(0.4, 7.6)	2.4	(0.9, 6.2)	1.9	(0.7, 5.3)
Exposure to intimate partner violence		2.3	(0.8, 6.4)	3.0	(0.9, 10.3)	5.0**	(2.1, 12.0)	6.0**	(2.6, 14.1)
No childhood maltreatment reported		0.7	(0.3, 1.6)	0.9	(0.4, 2.3)	2.7**	(1.6, 4.4)	3.1**	(1.8, 5.3)

Source: Statistics Canada, Canadian Community Health Survey -- Mental Health, 2012 (share file)

*CI* Confidence interval

^a^Adjusted for sex, income, education, race/ethnicity, and province

^b^Excluding immigrants to Canada

*Significantly different from reference group (*p* < 0.05)

**Significantly different from reference group (*p* < 0.01)

## Discussion

Ontario was the first province to introduce mandatory reporting legislation for CPA and CSA in 1965, quickly followed by Alberta and British Columbia. By 1981 all Canadian provinces had mandatory reporting for CPA and CSA. In 1973, Saskatchewan was the first province to mandate reporting of CEIPV. By 2014, all provinces except for Ontario and New Brunswick had enacted legislation requiring reporting of CEIPV.

Analysis of CCHS-MH retrospective data strongly suggests that the legal requirements to report suspected CM to child welfare services had an impact on CPO involvement. For those CCHS-MH respondents who had experienced CM, more of those who were born after 1965 reported having had contact with CPO than those born before mandatory reporting legislation was introduced in 1965. The influence of the 1965 legislation appears to have spread well beyond the borders of Ontario—to jurisdictions that had not yet introduced mandatory reporting. The subsequent introduction of legislation in other provinces and expansion of the mandate to report suspected CM likely further encouraged CPO involvement. Furthermore, the seriousness and frequency of maltreatment was directly related to the likelihood of CPO contact.

The importance of mandatory legislation to increasing the reporting of CM and identification of cases has been observed elsewhere. In Western Australia, reporting of CSA increased almost four-fold following establishment of mandatory reporting requirements [20]. Comparisons among countries with and without mandatory legislation also show higher reporting rates for jurisdictions with mandatory reporting than for those without [21]. The finding that the greatest increase occurred for those reporting the most frequent or serious types of CM is promising, especially in view of debates regarding over-reporting [22].

The merits of mandatory reporting have been debated [22–26], and some Canadian mental health professionals have called for revocation of such laws [27]. Opponents contend that mandatory reporting increases workload, is intrusive and diverts resources from assisting children and families in need [22, 28]. Melton, probably the strongest critic of mandatory reporting, argues that mandatory legislation was created based on a misunderstanding of the nature and scope of CM [22]. Other critics posit that child protection provides only an individual response to what is a broader, societal problem [29]. Advocates of mandatory reporting contend that reporting of CM to CPOs increase opportunities for beneficial intervention. Clients are generally satisfied with the interaction with a CPO [23], and those who report CM appreciate the laws related to privacy and confidentiality [30]. Furthermore, increased reporting to CPOs reflects government commitment and brings attention to the problem of CM [24].

The findings of this paper should be interpreted in light of its strengths and limitations. Strengths of this paper include the comprehensive doctrinal legal review that was employed. The statistical analysis used data from a large representative sample of Canadian adults. However, insufficient sample precluded more detailed analysis by specific types of maltreatment and province. Other limitations include the unknown validity of the CPO measure. It is possible that some respondents who reported CM were unaware that CPO involvement occurred or they did not recall the involvement. However, the finding that reported CPO involvement was rare among those who reported no CM provides some evidence of the validity of the CPO measure. The small proportion of respondents who reported CPO involvement but no CM may have experienced other types of CM not included in the CCHS MH such as neglect or emotional abuse. As well, reporting of CM may be subject to recall bias. However, evidence of the validity of retrospectively collected CM data is increasing [31–34]. Another limitation was the assumption that the respondent lived in the same province before age 16 as when the CCHS questionnaire was completed. Data from the 2011 National Household Survey indicate that 85% of the Canadian-born population resides in their province of birth [35]. As well, the study is limited to the household population in Canada’s 10 provinces and excludes the territories. Some groups who were not part of the CCHS target population (the homeless, residents of institutions, Indigenous peoples living on reserves, full-time military personnel and the northern territories) have in some studies been found to have elevated levels of CM [36–41]. How this might influence associations between CM and CPO involvement is unknown. Finally, it is possible that secular changes in social values, openness to acknowledge that CM has occurred, family characteristics or other factors may have increased reporting of CM to CPOs over time, independent of mandatory reporting laws.

Although it is undisputable that CM can have devastating immediate and long-term negative consequences, the effects of reporting to CPO are not well studied. Assuming that CPO reporting is related to less maltreatment and better outcomes for children, it is encouraging to see that increasing percentages of children who have experienced CM are being reported to CPO and that mandatory reporting has assisted in identification of children in need of protection. Unfortunately, this analysis does not inform our understanding about the effectiveness of CPO interventions themselves, such as the provision of services to parents and protective orders for children — an important area for future research [25]. Does contact with CPO and the measures taken by CPO reduce re-victimisation of children and prevent maltreatment of others? Do children get needed help to treat negative outcomes associated with their exposure to CM?

## Conclusions

This study compiled the history of mandatory reporting to CPO for CSA, CPA and CEIPV in Canadian provinces. The timing of these legislative changes was examined in relation to contacts with CPO as reported by survey respondents. Evidence from the analysis suggests that the legislation is effective in increasing CPO involvement. Further study should address if reporting to CPO is successful in preventing the recurrence of CM and providing effective assistance to victims and their families since its effectiveness as a public health strategy is unknown. The collection and analysis of longitudinal CPO data would significantly contribute to such efforts.

## Abbreviations

CCHS-MH: Canadian Community Health Survey-Mental Health
CEIPV: Childhood exposure to intimate partner violence
CEVQ: Childhood exposure to violence questionnaire
CI: Confidence interval
CM: Childhood maltreatment
CPA: Childhood physical abuse
CPO: Child protection organization
CSA: Childhood sexual abuse
LICO: Low income cutoffs

## Footnotes

1 This occurred where the term “sexual abuse” was sometimes not expressly included in the legal provision, but the term “ill-treatment or need for protection” was used, which would embrace sexual ill-treatment or the need for protection because of sexual abuse (and the perception in practice of this by the reporter). Verdun-Jones et al. [14] confirm that sexual abuse was often not expressly included in the initial versions of legislation, but may have been considered in practice as a species of physical ill-treatment, at least in cases of sexual abuse by parents or caregivers; and that subsequently the duty was expressly extended to sexual abuse. An example is Alberta, where the initial enactment of 1966 expressly applied to physical ill-treatment or need for protection, and the subsequent amendment in 1984 expressly included sexual abuse as a category of a child being in need of protection. Similarly, Quebec’s initial enactment applying expressly to physical abuse on 28 December 1974 (“physical ill-treatment as the result of abuse or neglect”) was enlarged by express inclusion of sexual abuse on 3 April 1984.
